# Uncoupled redox stress: how a temporal misalignment of redox-regulated processes and circadian rhythmicity exacerbates the stressed state

**DOI:** 10.1098/rsob.230151

**Published:** 2023-09-06

**Authors:** Anna D. Clark, Andrew F. Cumpstey, Jérôme Santolini, Alan A. Jackson, Martin Feelisch

**Affiliations:** ^1^ Perioperative and Critical Care Research Group, Southampton NIHR Biomedical Research Centre, University Hospital Southampton NHS Foundation Trust, Southampton SO16 6YD, UK; ^2^ Clinical and Experimental Sciences, Faculty of Medicine, University of Southampton, Southampton, SO16 6YD, UK; ^3^ Institute for Integrative Biology of the Cell (I2BC), CEA, CNRS, Université Paris-Sud, Universite Paris-Saclay, F-91198, Gif-sur-Yvette Cedex, France; ^4^ Human Nutrition, University of Southampton and University Hospital Southampton, Tremona Road, Southampton, SO16 6YD, UK

**Keywords:** stress, redox medicine, chronobiology, reactive species, Long COVID

## Abstract

Diurnal and seasonal rhythmicity, entrained by environmental and nutritional cues, is a vital part of all life on Earth operating at every level of organization; from individual cells, to multicellular organisms, whole ecosystems and societies. Redox processes are intrinsic to physiological function and circadian regulation, but how they are integrated with other regulatory processes at the whole-body level is poorly understood. Circadian misalignment triggered by a major stressor (e.g. viral infection with SARS-CoV-2) or recurring stressors of lesser magnitude such as shift work elicit a complex stress response that leads to desynchronization of metabolic processes. This in turn challenges the system's ability to achieve redox balance due to alterations in metabolic fluxes (redox rewiring). We infer that the emerging ‘alternative redox states' do not always revert readily to their evolved natural states; ‘Long COVID’ and other complex disorders of unknown aetiology are the clinical manifestations of such rearrangements. To better support and successfully manage bodily resilience to major stress and other redox challenges needs a clear perspective on the pattern of the hysteretic response for the interaction between the redox system and the circadian clock. Characterization of this system requires repeated (ideally continuous) recording of relevant clinical measures of the stress responses and whole-body redox state (temporal redox phenotyping). The human/animal body is a complex ‘system of systems’ with multi-level buffering capabilities, and it requires consideration of the wider dynamic context to identify a limited number of stress-markers suitable for routine clinical decision making. Systematically mapping the patterns and dynamics of redox biomarkers along the stressor/disease trajectory will provide an operational model of whole-body redox regulation/balance that can serve as basis for the identification of effective interventions which promote health by enhancing resilience.

## Graphical abstract

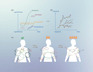

## 1. Introduction

The motions and periodicity of the solar system in which we live have shaped all life on Earth. The annual movement of the Earth around the sun, along with its tilted spin axis, establishes day length and seasons, which in turn influence patterns of food availability. The lunar cycle, set by the movement of the moon around the Earth and accounting for the gravitational ocean tides, accords with female fertility and reproductive capability while also affecting sleep quality. The daily rotation of the Earth on its axis establishes basic diurnal cycles evident in the entrainment of patterns of behaviour, metabolic function and cellular integrity. The solar energy captured in the form of the food that we consume periodically and the pattern of excretion of metabolic end products ensures that an appropriate energy and nutrient balance is established to maintain health. The exquisite sensitivity with which energy and nutrient balance are protected over long periods of time masks the periodic ‘ebb and flow’ of daytime feeding and night-time fasting. For all organisms including humans, the rhythm and pattern of turnover enabling this dynamic equilibrium has to be integrated at all levels of organization. Perturbations to this equilibrium have unfavourable consequences, leading to stress(es) of varying magnitude. Perturbations may derive from internal or external changes that challenge the homeostatic processes attempting to protect the ‘internal milieu’ and threaten the allostatic capacity. Challenges of sufficient severity are characterized as stress(ors). Coping with stressors through an adequate response is imperative for the preservation of health. Any mismatch or imbalance between the stressor and the response of a magnitude sufficient to exceed either the ability or the capacity to cope predisposes an organism to ill health. It can also manifest as maladaptation to extreme environments (polar regions, outer space). Resilience to environmental challenges is an expression of the buffering reserve in capability and/or capacity to protect and restore the integrity of the whole system with the recruitment of progressively higher levels of organization as needed (for a definition of the term ‘resilience’ see [1]). While most considerations discussed in this article focus on human health, the principles described hold true for all biological systems.

Almost all aquatic and terrestrial living systems on Earth ultimately derive their energy from the sun^1^ through processes that are intrinsic to the natural rhythms of Life itself, extending to habitats never exposed to natural sunlight [2,3]. The power derived from solar energy has enabled the evolution of many diverse life forms of great complexity associated with the emergence of novel capabilities. These capabilities provided the ability to cope with novel environmental challenges or metabolic stressors, with a response that proportionally matches their intensity and duration. Energy capture is readily identified as an increase in tissue mass (growth), but this energy not only enables the development, maturation and refinement of mechanisms that support growth and defend the metabolic integrity of the internal environment (homeostasis), it also supports the capacity to respond to internal or external stressors (allostasis). These capabilities have evolved over time from lower to higher and simple to more complex organisms. At all later stages, the structures and functions that provide the basis for these capabilities to respond build on earlier experiences. Perturbations in any aspect of periodicity may represent a challenge, which can exert effects at all levels of organization. When the perturbation is within the coping capacity it does not pose any threat to health, but when the stressor exceeds the coping capacity ill health supervenes. There may be modest changes of periodicity that are readily managed such as those brought on by crossing time zones by sea, but the response will be more intense with the acute and more severe changes during air travel. Similar physiological challenges are experienced with a shift from day-work to night-work or admission to the 24 h care provided within the intensive care unit of a hospital. These responses are readily definable, but over longer periods of time the chronicity of such low-intensity stresses can lead to the development of chronic non-communicable disease. The current transdisciplinary review suggests that these changes are manifestations of an uncoupling of entrained metabolic organization from wider environmental cues, mediated through disturbance of regulatory redox systems: perturbations in the circadian rhythmicity of the operation of the *Reactive Species Interactome* (RSI) that give rise to a new form of stress we here define as ‘*Uncoupled Redox Stress*'^2^.

The integrated response of acute illness also perturbs the metabolic rhythms of the body. This is exemplified by the ‘perfect storm’ elicited by the COVID-19 pandemic caused by human infection with the coronavirus SARS-CoV-2. This pandemic has exposed vulnerabilities at every level of biological and social organization; causing social disruption, emptying places that were bustling previously, killing millions of people and pushing healthcare systems beyond their limits. In order to further develop our understanding on how to manage COVID-19, we have explored the extent to which this vulnerability is a feature of an interplay between SARS-CoV-2 and reactive species, from the subcellular level to the whole organism [4]. Altered production of some reactive species had been observed at an early stage of the pandemic, often accompanied by alterations in the duration and quality of sleep, and mood changes. Yet, manifestations of the latter were also seen in uninfected healthcare personnel working night shifts [5], and in the population at large due to the unprecedented scope and scale of the societal restrictions [6]. Moreover, circadian disruptions had been observed before the pandemic in patients suffering from chronic pulmonary disease and other metabolic diseases such as diabetes and obesity [7,8]. Indeed, a direct molecular link between viral infection with SARS-CoV-2 and perturbation of cellular clock genes was established only very recently [9].

Circadian rhythmicity is a feature of body clocks, and clock genes are the hardwired link between environmental and metabolic periodicity. This invites consideration of whether there is a common underlying process through which these very different stressors could have disrupted natural rhythms in healthy individuals or in those with ill health. We propose that the uncoupling of bodily redox regulation from natural rhythms orchestrated by the circadian clock (*Uncoupled Redox Stress*) represents an important and previously unrecognized stressor in its own right that can exacerbate the potential harm caused by any other concurrent stressful experience. We briefly discuss the relevance of this new concept in the context of the current literature. In order to better refine the model of how stress signalling relates to whole-body redox regulation we further offer suggestions on the nature of this new form of ‘*Uncoupled Redox Stress*' and how best it might be studied by incorporating temporal considerations into future research. Finally, we explore the role redox systems might play in modulating resilience to further stress and susceptibility to disease.

## 2. Natural rhythms are intertwined with redox mechanisms

The importance of chronobiology, literally the study of time in living things, has been recognized since the eighteenth century [10]. The most obvious example of this is the circadian rhythm; others range from annual hibernation or migration to cellular and sub-cellular cycles. Much of life on Earth has evolved in the presence of diurnal/seasonal variations of light and temperature from which have emerged anticipatory systems geared towards maximizing chances for survival. Organisms can only thrive when they are able to adapt optimally to the periodic environmental cycles.

Circadian clocks are found in all prokaryotic and eukaryotic organisms. They have evolved from primordial systems responding to environmental cues. They vary in complexity from the most simple, with an ability to drive fluctuations in post-translational modification(s) of a single protein during the day/night cycle, to highly complex systems that comprise numerous oscillators that generate a robust, self-sustained circadian rhythm that can be fine-tuned (entrained) by external and metabolic cues [11]. In humans, the circadian rhythm is controlled centrally by a ‘master clock’ located in the suprachiasmatic nuclei (SCN) of the anterior hypothalamus [12]. However, there are many other ‘clocks’ elsewhere in the brain and in organs and cells of the periphery that can maintain their own 24 h oscillations independent of the ‘master clock’ for days [13]. How these different oscillators interact and harmonize in metazoans is not yet fully understood, but the network of oscillators is extensive and complex as virtually all cell types appear to have a clock mechanism [13,14]. A very recent study in mice illustrates the important role of inter-organ cooperativity of circadian clocks in achieving glucose tolerance, suggesting that a disruption of this fine-tuned spatio-temporal regulatory pathway contributes to circadian-rhythm-associated metabolic disease [15].

Redox signalling is a critical consideration for clock mechanisms. Redox-based cellular clock constituents including peroxiredoxins are found in many different species, and even exist in enucleated cells such as erythrocytes [16–21]. Peroxiredoxins are typically associated with antioxidative protection, stress resistance, ageing and longevity. However, one of the lesser-known functions of peroxiredoxins is the actual keeping of time. Not only have peroxiredoxins been evolutionarily conserved over millenia and across taxa [22], they also couple intermediary metabolism and cell division in an ultradian cycle [23]. Peroxiredoxins are believed to have emerged around the time of the Great Oxidation Event about 2.5 billion years ago [22], but on our planet the linkage between environmental redox changes and biology likely goes back to the origin of Life itself [24,25].

Redox reactions are involved in modulating clock mechanisms from the ‘master clock’ of the SCN, where rhythmically oscillating redox states affect SCN neuronal excitability [26], to individual cellular clocks in the periphery. Circadian clock genes have been found to modulate cell death in response to oxidative stress [27]; indeed, hydrogen peroxide (H_2_O_2_)-driven signalling causes a dose-dependent change in circadian clock phase in mouse embryonic fibroblasts *in vitro* and in mouse peripheral tissues *in vivo* [28]. Changes in intracellular redox states have been found to affect the circadian responses of mice via apoptosis signal-regulating kinase (ASK) signalling [29]. The reverse also seems to be true: in mice with dysfunctional circadian rhythm, H_2_O_2_ and Nox4 expression is increased, and Nox4 expression was found to oscillate in cell cultures [30]. The regulation appears to be mutually reciprocal, i.e. the ‘redox state’ regulates and is itself regulated by the circadian clock. This implies the ‘redox sweet-spot’ is constantly shifting, depending on the time of day. In addition, the same redox triggers may have opposite effects depending on the phase of the cellular clock or the time of day [31]. Along with the nature of this potentially bi-directional redox–clock interaction at the cellular level, there is the need to understand how the functional complexity that lies between the cellular and the whole-body level is organized and integrated. How can this ensure that natural rhythms are regulated to enable integration among different cells, the interstitium, within and between organs, and how does this determine whole-body redox state?

## 3. ‘Uncoupled redox stress': the stress caused by uncoupling redox signalling from natural rhythms

**Figure 1.**
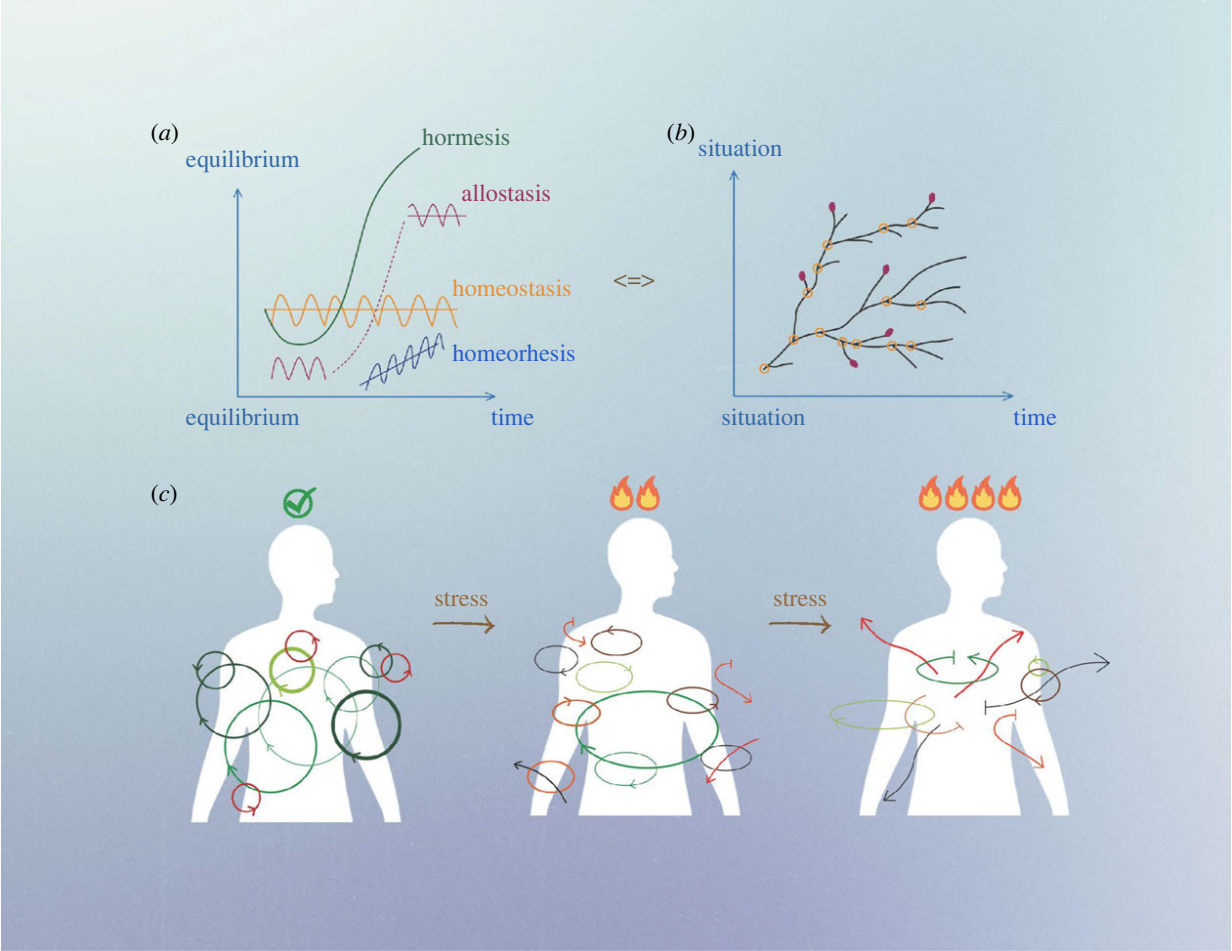
Panel (*a*) Evolution of (homeostasis-related) biological systems with time. Many biological regulation systems change over time (*hormesis*) while striving to maintain physiological states of equilibrium in response to changes in the internal or external environment. The set point of these equilibrium states can be constant (homeostasis), move with time (allostasis), or evolve gradually over time (*homeorhesis*). In biology, most processes show *hysteresis* such that a greater force is required to nudge the system to a different state than maintaining it at a given state and the systemic response lagging behind the driving force attempting to change its state (not shown). Panel (*b*) Evolution of biological systems due to processual and relational adaptation—a ‘Tree of life’. Systems evolve in response to different biochemical and ecological situations (aptation) with multiple trajectories. Instead of being defined by programmed processes related to a specific finality (teleological purpose; reaching a multi-level equilibrium), evolution of living entities results from the competition of multiple trajectories depending on kinetic performance and multi-layered physiological interactions. Orange open circles correspond to *stress* situations, marking ‘branching points’ where changes in environmental conditions and/or intrinsic functioning lead to development of multiple adaptative paths. Red closed circles correspond to trajectories that could not be supported internally or externally (dead-ends). The situation where we find ourselves now could easily have been at any other branching point. Organismal evolution is therefore a ‘Tree of Life’ with junctions, branches and extinctions, but also many different historical trajectories. Our inability to describe this evolution is due to a teleological bias (if it is like that, it is because it had to be like that) and a presumed impossibility of achieving a contrafactual evolution (there is only one timeline). Panel (*c*) Temporal synchronization of major biological and regulatory systems. Most biological processes obey circadian rhythmicity and are coupled via biological clock mechanisms. Colour code refers to coupling status (from green—coupled, to black—uncoupled). Arrow positions refer to synchronization status of each cycle with one another. Under standard conditions, most biological cycles are coupled ‘in phase’ (green), slightly ‘out of phase’ (different arrows) or ‘uncoupled’ (red circles). Increases in stress (in time and amplitude) challenge the integrity of the oscillator network and its coupling to redox-related processes, and more physiological and biochemical processes become gradually uncoupled, eventually causing desynchronization of major biological cycles.

Stress has often been characterized as a threat to a biological set point, a deviation that is then corrected by homeostatic mechanisms. However, the temporal variation in the nature of this set point, as described above, has received relatively little attention. The earlier focus on the concept of homeostasis, how the ‘milieu interior’ is controlled and regulated, has shifted to the idea of allostasis, the ability to respond to challenges and stressors, and hence the notion of ‘achieving stability through change’ [32,33] (figure 1). This invites exploration of what we have come to understand as stress and how we can develop an understanding that adequately accommodates the fluctuating nature of a dynamic homeostatic redox norm taking account of the biological rhythms intrinsic to Life.

Our current understanding of the concept of stress was derived largely as a characteristic of illness at the whole-body level, as initially described by Selye about 80 years ago [34,35]. This contrasts with characterization at the cellular level as described by Sies with the coining of the term ‘oxidative stress’. Selye used ‘stress’ to describe a ‘*non-specific response of the body to any demand’*. Implicit in this articulation was the notion of a graded response to life-threatening challenges, and for those stressors that did not lead to death there was an acquired ability that offered resilience against future exposure to a similar stressor. Similar considerations about the quality of the response to different degrees of stress or different stressors are currently explored in relation to research on oxidative stress [36–39], a considerable part of which relates to the cellular level. For a more detailed discussion about redox considerations at the whole-body level we refer the reader to a recent review [40].

Together, the approach of Selye's and Sies’ concepts contrast responses at the whole-body and cellular levels of organization. However, these concepts fail to adequately explain the complex multi-level interdependent nature of redox systems and stress responses. To better understand those interactions, we introduced the concept of the ‘*Reactive Species Interactome**’*** (RSI) [24]. When this conceptual framework was developed it did not formally embrace any consideration of the dynamic character of the temporal effects of natural rhythms, i.e. how the reactions and fluxes within the RSI wax and wane with circadian and other rhythms. Consideration of this introduces another dimension of complexity, albeit one that appears fundamental to the character and function of the RSI. The evidence suggests that potentially extracellular redox status plays a crucial role in keeping metabolism [40,41] and other bodily signalling processes aligned.

In larger metazoans, the extracellular fluid (lymph and plasma) is the medium that connects all cells and tissues with one another and thereby has the potential to play an important role as a ‘shared reference point’ for bodily redox regulation [24,40]. It follows that under usual circumstances the extracellular redox set points would be aligned with other bodily rhythms. This ‘expected state’ would make it possible for any misalignment or variability in whole-body redox status from circadian rhythmicity to be recognized as a deviation from normality that would feed back to trigger alarm/corrective actions. A functioning RSI is vital to cellular and organismal survival. Since redox processes are integral to clock mechanisms and themselves modulated by clock mechanisms, such uncoupling of redox regulation from natural rhythms would translate into a challenge to achieve bodily redox balance, which we here define as ‘*Uncoupled Redox Stress*'.

## 4. Consequences of ‘*Uncoupled Redox Stress*'

The short-term effects of uncoupled redox stress from natural rhythms are familiar as the fatigue, irritability and slowed mental processing experienced by those who have worked night shifts or suffered from jet lag. However, the symptomatic longer-term effects of the uncoupling may be less apparent. In 2019, the International Agency for Research on Cancer declared that night shift work was ‘probably carcinogenic to humans' [42]. More recent evidence suggests shift work is associated with increased risk of asthma [43], and it is well established that shift work is associated with increased cardiovascular disease and type 2 diabetes [44,45]; all these conditions are associated with oxidative stress, and indeed there is evidence that night shift work directly increases oxidative stress in healthcare workers [46]. Thus, it is plausible that frequent or recurring uncoupling from circadian rhythms is associated with alterations in redox regulation, with likely profound consequences for health.

‘*Uncoupled Redox Stress’* may be the common mechanism of a wide range of acute and sub-acute illnesses associated with the unspecific symptom of ‘feeling sick’ [35]. The importance of this became evident during the COVID-19 pandemic, which required large numbers of patients to be sedated and mechanically ventilated in the critical care environment. Alarming numbers of patients have been afflicted by the long-term sequelae of the acute infection also known as ‘Long COVID’ [47,48], which has all the characteristics of being the consequence of ‘*Uncoupled Redox Stress*'. The practical importance of this should not be underestimated. To the best of our knowledge, this problem has not been discussed in the literature, nor is it being considered in the healthcare setting. For patients, a combination of critical illness, sedation, artificial feeding regimens and lack of natural light, all of which are unavoidable features of the ICU environment, mutually reinforce each other to overwhelm bodily coping mechanisms, disrupt circadian rhythms, and in the worst cases can give rise to delirium [49–52]. There is some evidence for a beneficial effect from the administration of melatonin, an antioxidant hormone that is naturally produced and released into the bloodstream during the night with levels peaking in the early morning hours [50], but this is no ‘magic bullet’. Combined with the previously described redox dysregulation induced by viral infections and their post-acute sequelae [4], an added disruption of circadian rhythms that is tightly intertwined with redox status can only worsen an already dire situation.

In order to cope with growing numbers of ICU patients due to an ageing population (independent of any pandemic) there is a greater need for healthcare staff to work shifts, resulting in an increase in the numbers who experience circadian disruption and its associated consequences. The problem also extends to the wider population of non-healthcare workers in whom increased screen time (e.g. due to more frequent virtual communication) has affected established work routines well beyond the acute phase of the pandemic [51,52]. Whereas the impact of some of these alterations may not be realized fully for years to come it is evident by now that COVID-19 disrupted social interactions at a global level, perturbed production cycles with widespread economic consequences, and provided a major challenge for the functioning of society at large [53–55].

## 5. Relevance for future research

The construction and refinement of a conceptual framework such as the RSI is challenging, given the complexity of the component parts and the dynamic nature of the interactions at every level of organization, from the molecular or cellular to complex social constructs. However, it is a necessary step in the development of new approaches to the prevention and treatment of diseases in which the RSI plays a central role. This has to be the context of any consideration for ‘redox medicine’ if it has the goal of achieving ‘redox health’ for each individual at every step of the life course, which leads to improvement in the health of populations. Clearly, the idea of ‘*oxidative stress’* as a paradigm has become rather popular in clinical practice, but it is just one seemingly helpful facet of a much more complex construct. The danger of such oversimplification is that it has encouraged an erroneous impression that reactive oxygen species (ROS; and other oxidants) are largely associated with pathological processes. As a corollary, the identification of some disorders as ‘oxidative stress-related’ has given rise to the uncritical application of antioxidants as likely beneficial interventions. Clinical trials have failed to provide support for this approach, as more often than not the purported therapeutic value of antioxidants has not been realized in practice. Although the ‘oxidative stress’ concept has value and continues to evolve (e.g. by distinguishing eustress from distress; [36,38]), on its own it does not provide a sufficiently adequate understanding of how stress responses might operate at the whole-body level [37–40,56]. By the same token, measuring one-off markers of oxidative stress in isolation is unlikely to provide meaningful information on the redox state of the body. Not only is the RSI a complex ‘system of systems’ that goes well beyond ‘oxidation bad/antioxidants good’, it has to also incorporate an understanding of the temporal considerations discussed above. In order to address ‘*Uncoupled Redox Stress*' the nature of the relationships between circadian rhythmicity of redox processes and stress responses from the molecular/cellular to the whole-body level needs to be further elucidated. How does the system work? How are these processes synchronized across cell and organ systems? To what extent are they buffered to provide resilience?

To answer these questions requires an understanding of the hysteretic behaviour of these systems in health, as a reference for ill health. In order to identify the most suitable biomarkers of clinical utility there may need to be detailed, repeated or continuous measurements of an array of redox-active biomarkers across a range of ages and contexts. Characterizing the usual range and variability in the circadian pattern of the dynamic set for the redox system in healthy individuals is needed in order to interpret the hysteretic pattern of change with different degrees of ill health. This level of characterization of *‘Uncoupled Redox Stress’* in ill health will enable specific differentiation from the characteristic ‘ebb and flow’ dynamics in good health and its variability among individuals. There are innumerable markers that might be of interest and capture the redox state of different tissues, cellular or subcellular compartments. Comparatively little attention has been paid to the bioelectrical properties of the extracellular space. More than five decades ago, Shapiro pointed out the importance of the regulation of bodily redox status (E*_h_*) and the need to support the maintenance of whole-body redox balance in clinical practice [57]. He drew a comparison with pH, and the training of clinicians in the correct recognition of respiratory and/or metabolic acid-base imbalances and the appropriate support of patients. He suggested a direct equivalence in the recognition and treatment of perturbations of E*_h_*. While it is already possible to ‘redox phenotype’ individual patients along their disease trajectory for research purposes [58–60], relevant measures need to be developed for clinical practice. Exploration of how these might be applied in understanding the drivers for E*_h_* in different disorders at different stages of the hysteresis curve would make it possible to identify relatively simple interventions. Thus, in combination with circulating markers of metabolic, inflammatory and oxidative stress/damage markers a characterization of the extent of inter-individual variability in E*_h_* and other markers of whole-body redox state would provide the potential for a diagnostically driven, personalized approach to re-establishing a healthy redox balance.

Such systematic ‘redox phenotyping’ would allow the characterization of a functional model of bodily redox signalling. It would also enable rational interventions to prevent or treat ‘redox diseases’ which present as many complex chronic health conditions, including Long COVID, and have substantial global health and economic impact. Any redox state consonant with health is the resultant of the dynamic interactions within a complex system. The ability of the system to modify the dynamic interactions among component parts and the buffering capabilities intrinsic to resilience reflect nutrient and metabolic interchange in response to environmental challenge. While ‘metabolic reprogramming’ is perhaps best known in the context of cancer, it is an established feature of growth and differentiation in metazoans and a primordial response to various stressors across biological kingdoms and life forms. The associated alterations in metabolic fluxes reflect the attempts of the body to protect homeostatic acid/base balance and the expected redox status of cells and tissues. How interventions can be developed to support the system rather than driving it to further imbalance has to be approached with great care and clarity of purpose. Any intervention given at the wrong time or in the wrong place, has to recognize the mutual interaction between redox state and circadian rhythms, and take into consideration that the system probably operates in a fundamentally different manner in illness and in health. Empirical and epidemiological evidence indicate that interventions most likely to be effective in the chronic setting will centre around personalized medicine approaches to improving the buffering capacity and prevent *Uncoupled Redox Stress*, which include physical activity, sound nutrition, and mental wellbeing.

## 6. Conclusion

There is an imperative to develop and refine a conceptual framework for time-appropriate interventions that avoid provoking or exaggerating *Uncoupled Redox Stress*. Future research should determine integrated markers of stress states, indicative of multiple scales of redox signalling measured over time to capture the character of the dynamic system, such that the hysteretic pattern in ill health can be incorporated into routine clinical assessment and treatment in order to refine and improve patient care. If structured appropriately, this information can inform the development of an improved understanding of the health–disease continuum while enabling safe and constructive exploitation of the enormous potential the RSI framework holds to improve health outcomes for all.

## Data Availability

This work did not require ethical approval from a human subject or animal welfare committee.

## Endnotes

1 With exception of the biomass around hydrothermal vents, which is fuelled by chemical energy.

2 In this context, ‘uncoupled’ refers to a temporal misalignment of two or more processes from evolved natural rhythms and not to the phenomenon of ‘mitochondrial uncoupling’.

## Data accessibility

This article has no additional data.
